# Perspective: The promises of a holistic view of proteins—impact on antibody engineering and drug discovery

**DOI:** 10.1042/BSR20181958

**Published:** 2019-01-30

**Authors:** Ser-Xian Phua, Kwok-Fong Chan, Chinh Tran-To Su, Jun-Jie Poh, Samuel Ken-En Gan

**Affiliations:** 1 Bioinformatics Institute, Agency for Science, Technology and Research (A*STAR), Singapore; 2 APD SKEG Pte Ltd, Singapore; 3 p53 Laboratory, Agency for Science, Technology and Research (A*STAR), Singapore

**Keywords:** allostery, antibody engineering, drug discovery, reductionism

## Abstract

The reductionist approach is prevalent in biomedical science. However, increasing evidence now shows that biological systems cannot be simply considered as the sum of its parts. With experimental, technological, and computational advances, we can now do more than view parts in isolation, thus we propose that an increasing holistic view (where a protein is investigated as much as a whole as possible) is now timely. To further advocate this, we review and discuss several studies and applications involving allostery, where distant protein regions can cross-talk to influence functionality. Therefore, we believe that an increasing big picture approach holds great promise, particularly in the areas of antibody engineering and drug discovery in rational drug design.

## Introduction

Due to natural complexity and resource limitations such as those present in technical, computational, and experimental methods, the reductionist approach in biomedical science has often reduced proteins to a mere sum of its parts, namely subunits, domains/folds, secondary and super-secondary structure elements etc. As a result, scientists have been looking at proteins in parts based on domains and functional sites while ignoring the less characterized parts with no known functions. In some cases, new artificial classifications based on the reductionist approach were also introduced.

To date, the reductionist approaches in biomedical experiments provided significant insights into the predominant region(s) associated with specific functions. Such findings have, in turn, led to significant applications. For example, antibody fragments such as antigen-binding fragment (Fab), single-chain variable fragment (scFv), or Fc, are widely used as research reagents and as potential therapeutics [1,2], and the classifications of protein domains in structural refinement and functional predictions [3]. Yet, the reductionist approach, though amenable and highly useful, ignores the bigger picture of inter-regional communications and their possible co-operative effects [3] that would be useful for further detailed analysis.

Generally, protein domain cross-talks, coined here loosely as ‘allostery’, have largely been neglected due to the lack of whole structures for holistic investigations. Nonetheless, allostery is increasingly shown to be essential in manipulating protein functions, especially in the area of drug discovery such as designing allosteric drugs [4–8] to affect protein function by binding to distant pockets from the protein active site. Such allosteric effects have also been found in numerous proteins [9] such as aspartate carbamoyltransferase (ATCase) [10], bovine glutamate dehydrogenase (BGDH) [11], phosphofructokinase [12], and also in antibodies [13–18].

Therefore, given advances in technologies leading to advanced experimental and computational techniques in recent years, the next level of scientific breakthroughs may require looking at proteins as holistically as possible. Calls for such an approach are already present in various specialties [19–22] with these attempts aimed at putting together insights derived from reductionist investigations.

According to Regenmortel [19], revisiting biological systems wholly as systems is important [23]. On this line of thought, while limitations in looking at whole systems are ever present, we may, nonetheless, be already reaching a saturation point for scientific breakthroughs within the reductionist approach. Thus, we propose that it is now time to re-analyze proteins in their entirety (where possible). In this article, we will focus on the issues pertaining to computational structural analysis and the bottlenecks in translating them toward experimental and possible future clinical outcomes.

To further illustrate our point, we utilized augmented reality (AR) via the use of mobile apps (see commentary [24] for details on its methodology and usage).

## Antibodies and receptors

A resurgence of interest in the antibody and its regions is augmented by the 2018 Nobel Chemistry Prize awarded to Sir Gregory Winter for the ingenious phage display method that led to many antibody-based applications. In monomeric form, the whole antibody is known to be a Y-shaped molecule [25]. The two ends of the V-shaped variable (V) regions are for antigen recognition and binding. The stalk (constant or C-region), holding up the V-regions, binds and triggers immune effector cell functions via engagement of the Ig receptor [26]. Even though the antibody V(D)J recombination underlies the genetic system for antibody generation [27], structural and sequence analysis have led to an additional classification within the V-regions of frameworks (FWRs) and complementarity determining regions (CDRs), where the FWRs are scaffolds to hold up the CDR loops [28]. From this FWR-CDR classification, humanization of antibodies from rodent sources have been successfully performed, with some leading to therapeutics [29]. This technology of CDR grafting is however hindered by a high degree of trial and error given the lack of rule-based understanding. Different algorithms [30–33] do not fully agree with one another, often defining the boundaries of FWRs and CDRs differently, thereby requiring additional analysis (e.g. Ling et al. [15]) for reaffirmation.

**Figure 1:**
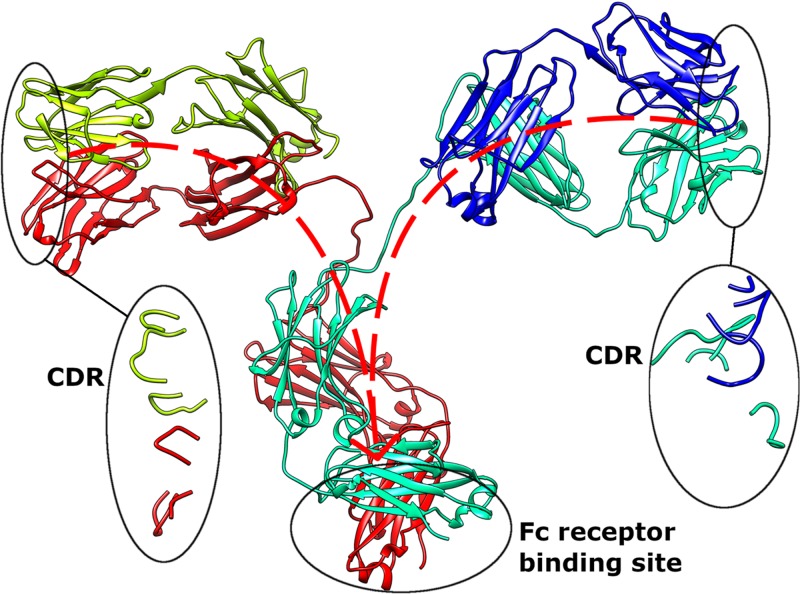
Visual representation of inter-domain signaling between CDR and Fc receptor binding region The antibody structure is retrieved from Protein Data Bank [25] (PDB: 1IGT). The animated (communication) effect can be viewed using the ‘APD AR Holistic Review’ app, available freely on Google and Apple app stores (view the image using the app camera, see commentary [24] for more details). Alternatively, download ‘HP Reveal’ from the stores and access the link, ‘http://auras.ma/s/wdpFQ’ to view the above image with the mobile app. An alternative video of the AR representation of this figure can be found at https://www.facebook.com/APDLab/videos/2075249849390855/.

Computational efforts to aid in *de novo* V-region design [34] hold great promise to synthesize antigen-specific antibodies from scratch, bypassing the contended animal-dependent methods. However, it is still essential to validate these *in silico* designs using *in vitro* experimental testing. There is no doubt that the structural classifications of CDR and FWRs are useful; however, neither CDRs nor FWRs alone can yield significant outcomes in isolation. In fact, recent evidence have demonstrated the interdependency of FWRs and CDRs in the binding of antigens, antibody production and purification, and even the functioning of distal antigen-binding regions [15]. To complicate things further, the C-region, typically neglected in experimental affinity maturation experiments relying on scFv or Fabs [35], can affect antigen binding as well [14,36]. Such findings highlight the need to also study the less studied antibody allotypes [37]. At the same time, the V-region FWR families of both antibody heavy (VH) and light (VL) chains were also found to affect C-region receptor binding [15], possibly modulating effector cell functions [38] (Figure 1).

Many reports [13,15–17,39] have demonstrated allosteric communications between various antibody domains (C-region and the antigen-binding regions) in IgG antibodies. Yang et al. [16] showed the allosteric co-operativity of both the V- and C-regions, rationalizing the structure–function relationship to go beyond the conventional domain-based hypothesis. In other antibody isotypes, similar findings to IgG [15,17] were also reported. Lua et al. [36] demonstrated changes in antigen engagement, where the same V-regions had equilbirum dissociation constants that indicated stronger (for IgM, due to avidity effects) or weaker (for monomeric IgD, and IgA and its subtypes) interactions by simply changing the heavy chain C-regions alone [36]. This effect was however not found when the light-chain C-regions were swapped. A follow-up study [14] focussing only on IgA further demonstrated that the allosteric signaling propagated bidirectionally between the V- and C-regions via the domain-linking hinge.

In the case of antibody-dependent enhancement (ADE), the antibody–receptor interaction-mediated endocytosis enhanced the infection of the dengue virus [40,41], which would likely be avoided by using the high avidity IgM instead [42]. Similarly in studies of other infectious diseases such as HIV [43], a systems level investigation (a holistic view) on ADE, where antibody therapeutics can be engineered not only to optimize the interaction with other molecules, but also to strike a balance between the efficacy of the drug and unwanted effects, would be important. Certainly, considering the molecule as a whole is useful especially when developing therapeutic antibodies, in which communication between the antibody and antigen/receptor play a key role. In the light of such effects across antibody regions, there is a reason to expect that detailed understanding and application requires the consideration of the whole antibody engagement to the antigen and/or Fc receptor.

Beyond antibodies, antibody receptors have also defied the reductionist-based approach where certain sequence regions exert effects beyond their boundaries. One such example is the IgA Fc receptor FcαR (CD89). The natural variant of this receptor molecule contains a full signal peptide and extracellular (EC) domains that bind to IgA antibody. Lua et al. [44] discovered that when a natural variant of the receptor lacking only the EC1 domain responsible for binding the IgA molecule [44] but having the full signal peptide was studied, the variant was found spatially constrained intracellularly rather than extracellularly. Attempts to ‘force’ EC localization, using other secretory signal peptides and mutations at the signal peptide cleavage sites, yielded no success [44]. Further studying other variants (in the presence of the EC1 domain and the complete signal peptide) showed that the lack of the other EC domain, EC2 located more distantly from the signal peptide than EC1, also prevented the EC localization [45]. It may suggest that for proper localization, all EC domains of CD89 are required to be present despite their distance from the signal peptide, demonstrating that protein domain co-operation is more enigmatic than expected.

In the investigation of antibody–receptor interaction, Ling et al. [15] found that different VH-VL FWRs of IgG1 variants, with the same CDRs, exhibited different equilibrium dissociation constants to the FcγIIA IgG receptor as well differently to the antigen. In several VH–VL FWR combinations, there were compromised FcγIIA interactions but not to the antigen [15]. This raises questions if neglecting Fc receptor engagement can result in therapeutic antibodies with reduced immune effector cell engagement. Especially when further analyses demonstrated that by varying the VL pairs (not involved in direct FcR binding), FcγIIA equilibrium dissociation constants could be reinstated to that of the control antibody without compromising antigen equilibrium dissociation constants. While the underlying mechanisms of such effects are still elusive, further investigations would need to take a more holistic approach involving whole Ig–FcR complexes.

## Allostery for druggable targets and drug discovery

The use of allostery for druggable targets in intervening pathogenesis of many diseases are many, ranging from: (i) identifying allosteric targets that influence enzymatic activity; (ii) identifying allosteric epitopes/sites for targetting by antibodies [46–49] and/or inhibitors [50–55] to affect the active site; or (iii) repurposing existing natural enzymes.

### Allosteric targets that influence enzymatic activity

**Figure 2:**
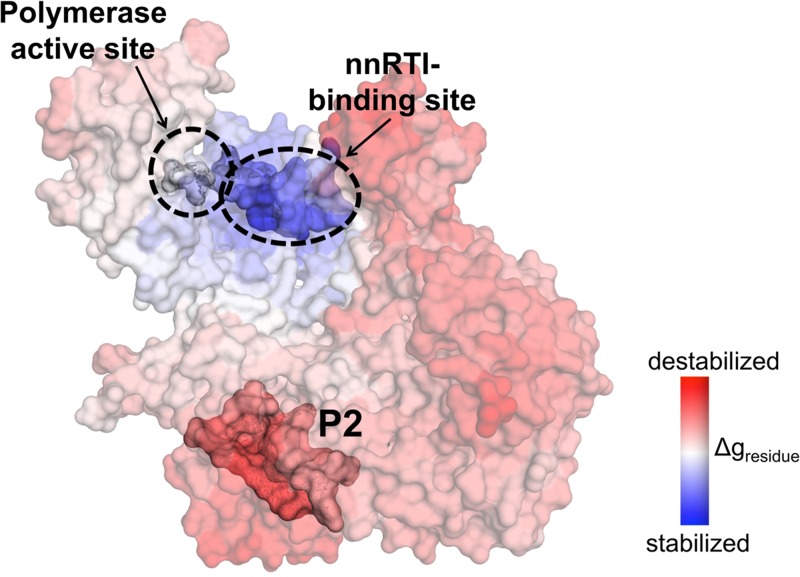
Allosteric communication of Pocket P2 on the polymerase active site This figure shows the rigidifying effects caused by the potential druggable pocket P2 on the polymerase active site (blue), subsequently regulating the function of the enzyme. Figure adapted from Chiang et al. [56]. An animation of inhibitor binding to the allosteric NNRTI-binding site can be viewed using the ‘APD AR Holistic Review’ app, available freely on Google and Apple app stores (view the image using the app camera, see commentary [24] for more details). Alternatively, download ‘HP Reveal’ from the stores and access the link, ‘http://auras.ma/s/wdpFQ’ to view the above image with the mobile app. An alternative video of the AR representation of this figure can be found at https://www.facebook.com/APDLab/videos/2075249849390855/. Permission to use this figure has been granted by the authors.

In HIV therapy, promising results were achieved in the search for druggable pockets and potential alternative inhibitors to inhibit viral enzymes, particularly HIV-1 Reverse Transcriptase (RT) [56]. Of the two current classes of RT inhibitors (RTIs), the allosteric non-nucleoside RTIs (NNRTIs) target an allosteric pocket located 10 Å away from the polymerase active site and disrupted the active site. By studying the full structure of RT, alternative allosteric pockets away from the active site, can be identified for drug targetting [56, 57]. For example, Chiang et al. [56] performed virtual screenings for alternate allosteric pockets using AlloPred [58] and AlloSigMA [59] and detected three such pockets located on p51 subunit (but not on the active site subunit p66, shown in Figure 2). Given the increasing reports of HIV drug resistance [60] to the current Highly Active Antiretroviral Therapy (HAART), the novel allosteric sites on the RT p51 subunit opens up opportunities for novel drug sites, where various screening methods [61] such as ligand-based or structure-based virtual screening [62], can be applied.

### Allosteric targets for antibodies or inhibitors

**Figure 3:**
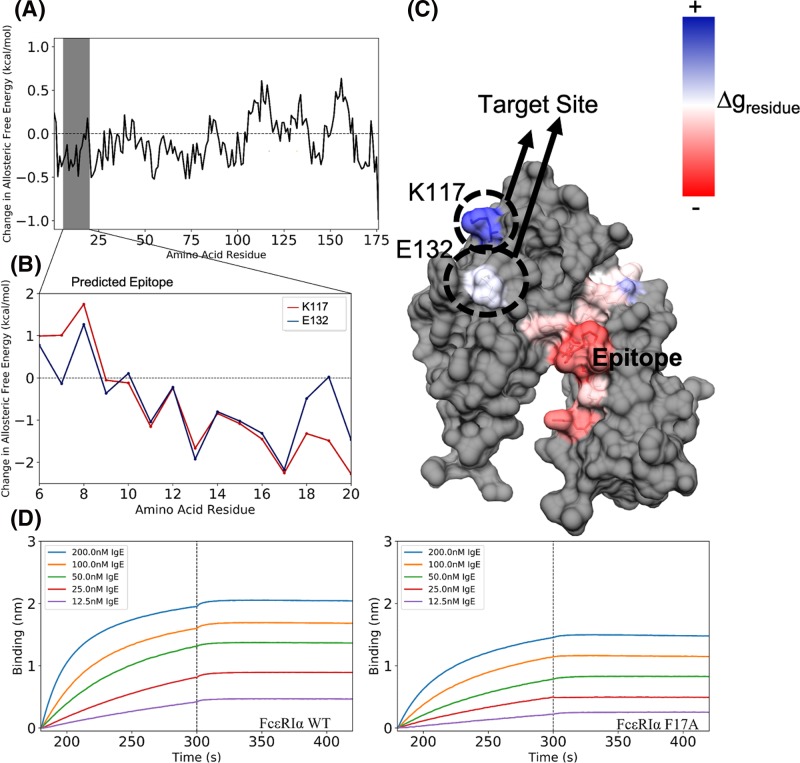
Analysis of allosteric communications between the identified epitope and the FcεRIα active site (**A**) Allosteric responses at each FcεRIα residue (X-axis) in the event of IgE binding (i.e. the active site residues K117 and E132, previously identified by Cook et al. [87], were assigned as ‘site’ in the AlloSigMA server [59] using the FcεRIα structure PDB: 1F2Q). The predicted epitope region (from K6 to E20) is highlighted in gray. (**B**) Estimated allosteric effects on each of the two active site residues by the individual mutation on the predicted epitope (i.e. each residue of the epitope was assigned at ‘UP-mutation’ in the AlloSigMA server). (**C**) Structural representation of allosteric communication between the identified epitope region and the IgE-FcεRIα interacting site. (**D**) Bio-layer interferometry binding experiments of the FcεRIα wild-type (left) and the F17A FcεRIα mutant (right) to the IgE at different concentrations. The animated (communication) effect can be viewed using the ‘APD AR Holistic Review’ app, available freely on Google and Apple app stores (view the image using the app camera, see commentary [24] for more details). Alternatively, download ‘HP Reveal’ from the stores and access the link, ‘http://auras.ma/s/wdpFQ’ to view the above image with the mobile app. An alternative video of the AR representation of this figure can be found at https://www.facebook.com/APDLab/videos/2075249849390855/

In the application toward allergy treatment, there is great promise to disrupt the IgE antibody and IgE Fc receptor (FcεRIα) interaction. Here, the identification of potential allosteric drug target sites using computational epitope and allosteric analyses (Figure 3A) can be performed. First, we performed epitope prediction using BepiPred-2.0 [63], Emini Surface Accessibility [64], and ABCPred [65] on the EC FcεRIα structure [66] (PDB: 1F2Q) followed by quantitating the allosteric communications between the individual residues of the predicted epitopes and the IgE FcεRIα-binding site using AlloSigMA [59]. We found the residue positions W13 and F17 on the FcεRIα to be potential mutation targets (Figure 3B,C). In the process of making both the mutants and the wild-type control using site-directed mutagenesis and transient transfection methods [15,36,44,67], the W13A mutant could not be produced at detectable amounts and therefore could not be subjected to subsequent experiments. Results of bio-layer interferometry (using nickel-NTA biosensors to capture the purified FcεRIα proteins followed by interacting with IgE at various concentrations from 200 to 12.5 nM) shown in Figure 3D demonstrated that the F17A mutation experimentally reduced the IgE-FcεRIα responses. This finding is consistent with a previous study by Mackay et al. [68]. Our combined methodology of *in silico* prediction and *in vitro* validation took a few weeks and were able to reproduce the previous conclusion, thus demonstrating a simplified process without tedious sequential single mutation experiments to find and validate such allosteric epitopes. Given that there would generally be a higher number of allosteric sites to target than the active site alone [69], such an approach allows additional allosteric epitopes to be identified in a wide range of proteins, as well as the potential for reverse perturbation to fine-tune and target allosteric responses [70].

Taking a step further, allostery-induced changes can include the more roundabout way to expose buried epitopes that would enhance immune detection. By studying the dynamics of the whole protein, buried binding sites can be exposed. In one example, Fuentes et al. [71] found a Trastuzumab-induced ‘cryptic epitope’ on Her2 that enhanced Pertuzumab interaction in simulations. Although this effect was not shown in *in vitro* experiments [72], such an approach is worth pursuing in proteins with no clear epitopes, allosteric or otherwise. Online databases such as SYFPEITHI [73], BIMAS [74], IEDB [75], and other such allosteric site prediction servers can help reveal buried epitopes and potential allosteric sites on the protein. This indirect approach may benefit from the combinatorial uses of small molecule inhibitors together with biologics to expose drug relevant sites/pockets/epitopes.

Nonetheless, the nature of allosteric inhibitors and therapeutic targets can be a double-edged sword. On one side of the blade, allosteric sites, having no intrinsic function of its own, may not be conserved and be easily mutated without affecting core functions of the proteins. As a result, resistance against therapeutic agents might develop quickly with mutations occurring directly on or between the allosteric site and the functional site to disrupt the allosteric communication. While it is possible to overcome the rapidly emerging mutations by screening for structurally conserved regions, this is conceptual at this point.

On the other hand, lack of direct inhibition on the active site may also allow for reduced inhibitory flexibility. The flexible nature of numerous proteins may result in partial rather than complete inhibition (see above FcεRIα example). It is advantageous to lower the selection pressure for drug resistance that may allow allosteric drugs to last longer. In addition, the partial inhibition may also allow the immune system to deal naturally with diseases that may be more beneficial in view of immune memory and natural resilience. Admittedly, this discussion of the potential uses of allosteric biologics and drugs is merely conjecture at this point, for even in the very established HIV therapy, NNRTIs are yet to reap the proposed benefits. While HIV is likely to be unique in its adaptive ability that other infectious agents or cancers are unlikely to follow, further work is certainly required.

### Allosteric targets to repurpose natural enzymes

The repurposing of natural enzymes against disease agents by allosteric mutations to affect the catalytic site is the final discussed approach to use allostery in unique ways beyond that of allosteric epitopes or druggable pockets.

A proof-of-concept to this approach was found in the insulin degrading enzyme (IDE) that was modified to have catalytic activity toward an amyloid β peptide [76]. Since the target modifications were performed on natural enzymes, chances of eliciting unwanted immune responses were low. However, in those cases, it is perhaps wise to avoid sites on the protein surface to reduce immunogenicity.

**Figure 4:**
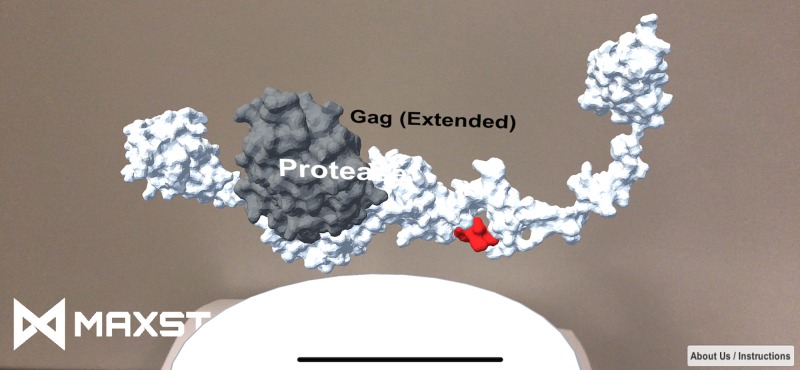
AR illustration of the extended conformation of the HIV-1 Gag polyprotein More details can be explored using the ‘APD AR Holistic Review’ app, available on Google and Apple app stores (pointing the running app on to Figure 2 in Su et al. [77] as the target image). Alternatively, download ‘HP Reveal’ from the stores and access the link, ‘http://auras.ma/s/wdpFQ’ to view Figure 2 in Su et al. [77] as the target image with the mobile app. An alternative video of the AR representation of this figure can be found at https://www.facebook.com/APDLab/videos/2075249849390855/

In viral infections, structural modeling of the whole HIV Gag protein [77] provided functional insights into a neglected Gag domain – p6 – for potential intervention in viral maturation (Figure 4). While p6 might not be easily drugged due to its high flexibility, analysis of the full-length Gag structure showed its contribution to the Gag conformational changes during maturation. Should there be intracellular interacting partners that could be engineered to constrain p6, viral maturation could be antagonized. Also, smaller antibody fragments or other repurposed proteins could be potential p6-binding candidates.

There is a clear need for more intensive research in these areas, and such efforts promise to generate more novel biologics against a variety of diseases, particularly those involving protein aggregation and viral infections.

## Challenges in considering proteins as whole

As discussed in several examples above, allostery is represented by communication effects between distal regions of proteins. Various allosteric models have been proposed, e.g. from the classical MWC model involving dynamics couplings of protein conformational changes [78] that interconvert concertedly, to the sequential KNF model where conformations of the involved domains sequentially change one at a time [79] leading to the propagation of the changes [80]. One of the most recent proposals argue the underlying allosteric mechanism to be derived from the population shift of the protein conformational ensembles, in which the more predominant conformational state drives the protein function [81–83]. Nonetheless, all the models imply the engagement of the whole protein structure, which otherwise would have been biased in any absence of the involved partners.

Such efforts are challenging when applied to a large system, e.g. antibody, multi-domain proteins, or membrane proteins etc., and when the structures of interest are far from achievable given current experimental limitations or computational bottlenecks. Besides that, ‘the elephant in the room’ is the poor translatability of computer predictions to experimental observations. Apart from constraints in computing resources that have gradually been resolved with technological advances, novel insights can come from considering whole proteins with multi-scale simulations and modeling. As computing power improves, the microenvironment can be included, e.g. entire viral organisms [84]. Otherwise, the availability of coarse-grained approaches [58,59,85] certainly can be a possible alternative.

Nonetheless, as discussed in the above examples, the approach of looking at biomolecules holistically allows the study of allosteric communication and allostery-derived interventions. Allostery is likely to apply to all types of proteins [83,86], promising to identify novel druggable sites, pockets, and repurposing enzymatic/binding activity. On this basis, considering whole proteins would be beneficial in detecting more allosteric sites, and also in providing new understanding of the subject matter.

## Conclusion

We are not pushing for extreme holism as warned by Regenmortel [19], which would be ineffective given current real-life resource constraints. While the reductionist approach still has great value, moving toward a more holistic approach in considering whole proteins, protein complexes, and potential microenvironments would certainly be useful, at least in the areas of antibody engineering, druggable targets, drug discovery, and enzyme repurposing.

## Abbreviations

ADE: Antibody-dependent enhancement
CDR: Complementarity determining region
EC: Extracellular
Fab: Antigen-binding fragment
FWR: Framework region
NNRTI: Non-nucleoside reverse transcriptase inhibitor
RT: Reverse transcriptase
RTI: Reverse Transcriptase inhibitor
scFv: single-chain variable fragment
